# Effects of Motor Imagery and Visual Neurofeedback on Activation in the Swallowing Network: A Real-Time fMRI Study

**DOI:** 10.1007/s00455-019-09985-w

**Published:** 2019-02-15

**Authors:** Silvia Erika Kober, Doris Grössinger, Guilherme Wood

**Affiliations:** 1 Institute of Psychology, University of Graz, Universitaetsplatz 2/III, 8010 Graz, Austria; 2 BioTechMed-Graz, Graz, Austria

**Keywords:** Deglutition, Deglutition disorders, Dysphagia, Motor execution, Motor imagery, Swallowing, Real-time fMRI

## Abstract

Motor imagery of movements is used as mental strategy in neurofeedback applications to gain voluntary control over activity in motor areas of the brain. In the present functional magnetic resonance imaging (fMRI) study, we first addressed the question whether motor imagery and execution of swallowing activate comparable brain areas, which has been already proven for hand and foot movements. Prior near-infrared spectroscopy (NIRS) studies provide evidence that this is the case in the outer layer of the cortex. With the present fMRI study, we want to expand these prior NIRS findings to the whole brain. Second, we used motor imagery of swallowing as mental strategy during visual neurofeedback to investigate whether one can learn to modulate voluntarily activity in brain regions, which are associated with active swallowing, using real-time fMRI. Eleven healthy adults performed one offline session, in which they executed swallowing movements and imagined swallowing on command during fMRI scanning. Based on this functional localizer task, we identified brain areas active during both tasks and defined individually regions for feedback. During the second session, participants performed two real-time fMRI neurofeedback runs (each run comprised 10 motor imagery trials), in which they should increase voluntarily the activity in the left precentral gyrus by means of motor imagery of swallowing while receiving visual feedback (the visual feedback depicted one’s own fMRI signal changes in real-time). Motor execution and imagery of swallowing activated a comparable network of brain areas including the bilateral pre- and postcentral gyrus, inferior frontal gyrus, basal ganglia, insula, SMA, and the cerebellum compared to a resting condition. During neurofeedback training, participants were able to increase the activity in the feedback region (left lateral precentral gyrus) but also in other brain regions, which are generally active during swallowing, compared to the motor imagery offline task. Our results indicate that motor imagery of swallowing is an adequate mental strategy to activate the swallowing network of the whole brain, which might be useful for future treatments of swallowing disorders.

## Introduction

Swallowing is a complex motor behavior that requires voluntary movements as well as involuntary reflexes [1]. The swallowing process activates a network of brain regions including the bilateral sensorimotor cortex, primary motor, premotor cortex, supplementary motor area (SMA), insula, cingulate gyrus, inferior frontal gyrus, inferior parietal lobule, temporal lobe, precuneus, basal ganglia, the cerebellum, and brain stem [2–7]. Since successful swallowing involves such a large neuronal network, different brain lesions lead to difficulties in swallowing or dysphagia [8]. Dysphagia symptoms reduce quality of life and health of affected people dramatically [8–10]. Up to 65% of neurologic patients show dysphagia symptoms. There is also a normal, age-related decrease in swallowing function. Up to 16% of neurologically healthy elderly people show swallowing difficulties [8, 10–12]. Traditional therapeutic approaches to treat symptoms of dysphagia include the modification of food or fluid consistency, external stimulation (e.g., massage and electrical stimulation) of oral and pharyngeal structures, feeding assistance, mealtime supervision, tube feeding, or parenteral nutrition [13–15]. However, it remains unclear whether neurologic patients who managed to use these techniques fare better than those receiving no specific dysphagia therapy and compliance to treatment is generally low [13].

In this context, neurofeedback (NF) might be an alternative and innovative treatment tool for dysphagia. In general, in NF applications, brain signals are recorded with different neuroscientific methods [electroencephalography (EEG), magnetoencephalography (MEG), functional magnetic resonance imaging (fMRI), near-infrared spectroscopy (NIRS)], processed in real time by a computer and fed back to the NF user online via visual, auditory, and/or tactile feedback. Thereby, NF users can “see” directly what is happing in specific brain regions while performing specific tasks. Consequently, they can learn to modulate voluntarily their own brain activity, which can lead to functional improvement [16, 17]. In the context of dysphagia rehabilitation, NF can be used to provide real-time feedback about the level of activation in brain areas, which are involved in the swallowing process addressing directly the neuronal underpinnings of swallowing. In this context, prior biofeedback studies providing feedback of physiological signals [electromyography (EMG), accelerometer, signal assessed by an oral pressure-monitoring device] during active swallowing could show that receiving real-time feedback of the swallowing process has beneficial effects on swallowing function [18–24]. Therefore, we are confident that receiving real-time feedback of one’s own brain signals during swallowing using NF might have positive effects on the swallowing function, too.

Receiving feedback about the activity in motor areas can foster neuronal plasticity and consequently improve motor functions [25–34]. This has been constantly proven for limb movements. In prior NF studies, participants imagined to move their hand or foot, which generally led to comparable brain activation patterns than executing these movements [25, 35–41]. In this context, motor imagery (MI), which is defined as the mental imagination of a specific motor act without overt movements by muscular activity, is often used as mental strategy in NF applications [39–41]. NF studies showed that healthy individuals as well as neurologic patients are able to learn to modulate voluntarily the activity in motor brain areas by means of MI strategies [25, 29, 30, 32, 35–38, 42].

It is an open question whether ME and MI of swallowing also activate similar networks in the brain. On the one hand, the quality of swallowing movements cannot be assessed easily from outside, and its diagnostics requires endoscopic or videofluorographic exams [43], so that in contrast to movements of the limbs, the only form of feedback on swallowing movements someone naturally can rely upon in real life is kinesthetic. On the other hand, only a few near-infrared spectroscopy (NIRS) studies directly compared brain activation patterns during MI and ME of swallowing [44–47]. These studies found overlapping activity in the inferior frontal gyrus and precentral regions (including premotor areas and SMA) between both tasks [46]. Although the temporal resolution of NIRS is higher as the temporal resolution of fMRI and NIRS is a portable, cheap, and easy-to-use neuroimaging technique enabling neuroscientific measurements in clinics and institutions where no MRI scanner is available, the spatial resolution of NIRS is not as high as of fMRI. With NIRS, only relative concentration changes in the hemodynamic response in the outer layer of the gray matter of the brain can be assessed, but not in deeper brain areas such as the basal ganglia, insula, or brain stem [48, 49]. Since active swallowing also leads to activation changes in deeper brain regions [2–4, 6], fMRI might reveal a more profound picture of neuronal mechanism underlying MI and ME of swallowing. Prior fMRI studies investigated ME and MI of mouth movements, only [40]. When using MI of swallowing as a mental strategy in NF applications, it is essential to prove that MI activates brain regions comparable to those during ME of swallowing.

The aim of the present study was twofold. First, we investigated whether execution of swallowing movements leads to comparable brain activation patterns than those elicited by imagery of swallowing movements. We expect that both tasks lead to comparable activation patterns, especially in the inferior frontal gyrus and lateral precentral areas [44–46]. However, we also expect that by using fMRI, we will observe comparable brain activation patterns between both tasks in deeper brain areas, which cannot be assessed with NIRS, such as the insula, cingulate gyrus, basal ganglia, or the cerebellum [2–6]. According to prior fMRI studies, we expect activation patterns in the sensorimotor cortex, primary motor, premotor cortex, and SMA during both swallowing tasks, too [2–6].

Second, we wanted to know whether healthy subjects are able to modulate voluntarily their brain activity in areas activated during MI of swallowing. Therefore, we used a real-time fMRI paradigm to provide participants with visual feedback about their activation level in brain areas involved in swallowing. We hypothesize that activation in brain areas, which are involved in the swallowing process, should be higher during NF training than during an offline task, in which participants imagined swallowing movements but did not receive real-time feedback about their own brain activity [37, 45].

## Methods

### Participants

Eleven right-handed, healthy young adults (4 male, 7 female, mean age = 29.18 years, *SD* = 5.62) took part in this study. All participants gave written informed consent. They had normal or corrected-to-normal vision and no history of major medical illness, neurological, or psychiatric disorders. None of the participants had a history of swallowing difficulties or a difficulty in language comprehension. The study was approved by the Ethics Committee of the University of Graz, Austria (reference number GZ. 39/69/63 ex 2016/17) and is in accordance with the ethical standards of the Declaration of Helsinki.

### Study Design

**Fig. 1:**
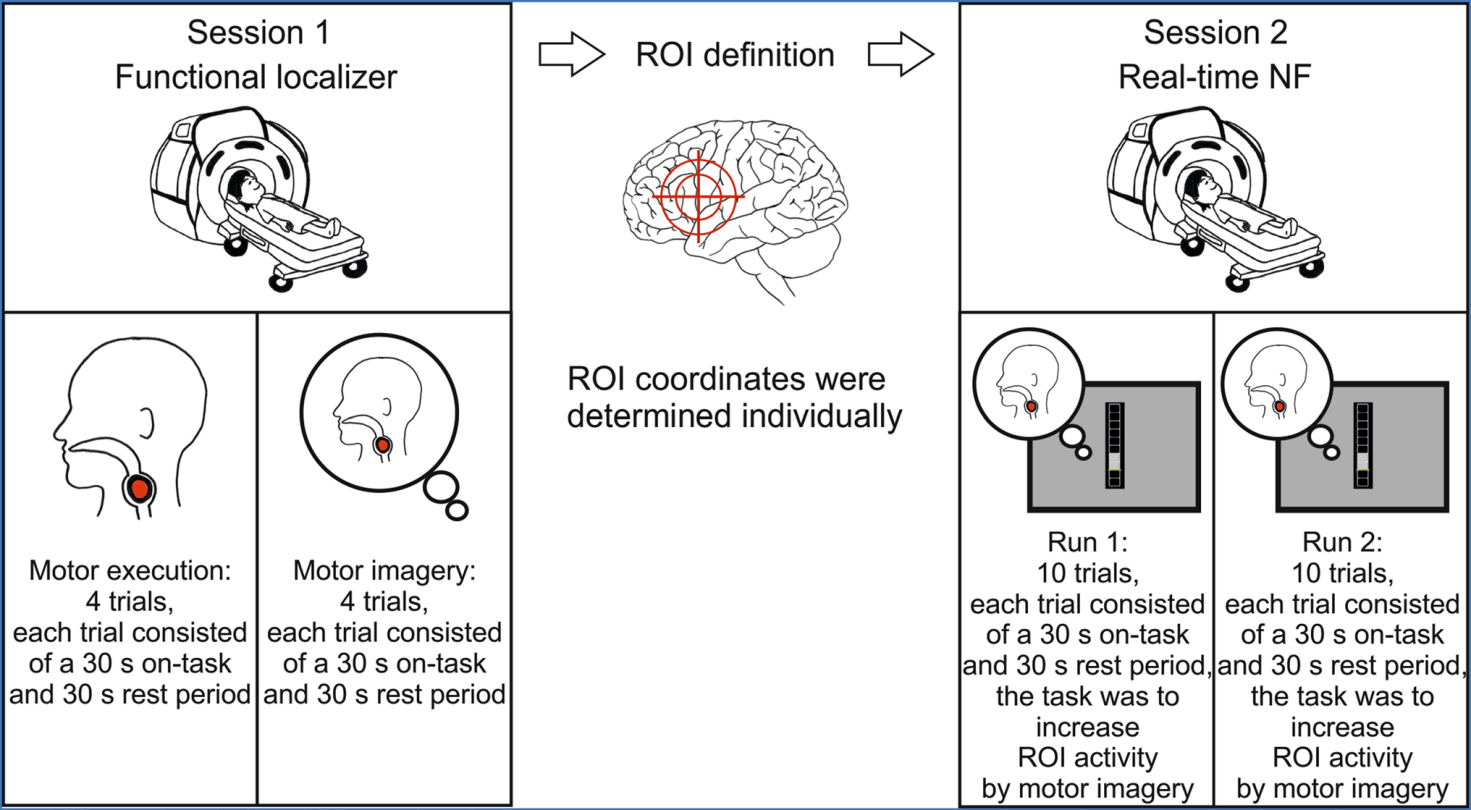
Design of the study. ROI: region of interest

All participants performed two MRI sessions on two different days. During the first session, participants performed a functional localizer task to identify brain areas, which are active during executing and imagining swallowing movements. No real-time feedback was provided during this first session. The data recorded during the functional localizer task was analyzed offline and used to determine the individual region of interest (ROI) coordinates for the second session, in which participants performed a real-time NF task. During the NF task, participants received visual feedback (a thermometer bar changed its size) of activation changes in the feedback ROI. The aim of the participants was to increase the activity in the feedback ROI as indicated by the size of the thermometer bar by imagining swallowing movements. The functional localizer task and the NF task are described in more detail below. Figure 1 illustrates the study design.

### Session 1: Functional Localizer

During the first session, participants performed the functional localizer task to identify brain areas, which are active during MI and ME of swallowing. Before participants laid in the scanner, written instructions about the task and the whole procedure were provided. Participants were instructed to swallow repeatedly in a regular, comfortable, self-paced rhythm during the 30 s ME trials, which resulted in about 5–6 swallows per trial. Note that the exact amount of swallows per ME trial was not recorded. Participants were instructed that each ME trial would take 30 s and that they should swallow in the same rhythm for the whole duration of the trial. They should stop swallowing when a written instruction presented on the screen indicated the start of the pause interval. For the MI task, participants were instructed to imagine swallowing in the same rhythm as during the ME task. They also executed and imagined the swallowing movements before the MRI task. Together with the experimenter, execution of repeated swallowing movements (5–6 swallows over a period of 30 s) and subsequent imagery of the swallowing movements was performed before lying into the scanner.

During the MRI measurement, a T1 scan was performed first. Then, the functional localizer task started. This task consisted of 4 ME trials and 4 MI trials. Each trial lasted 60 s, consisting of a 30 s task period (either ME or MI) and a subsequent 30 s rest period. During the first 3 s of the task period, a written instruction was presented on the screen (“*Please start swallowing*”, “*Please start imagining to perform a swallowing movement*”), indicating the start of the task period, followed by a fixation cross. During the first 3 s of the rest period, a written instruction presented on the screen indicated the start of this pause interval, in which participants were instructed to relax and to avoid active swallowing movements. The written instruction was followed again by a fixation cross for the rest of the pause interval.

Participants were instructed to swallow saliva as long as the ME task interval lasted. During the MI task, participants were instructed to imagine how it feels (kinesthetic imagery, [50]) to swallow for the whole task duration and to avoid active swallowing movements. There is evidence form EEG studies that kinesthetic MI leads to a stronger activation of motor areas, which are also active during motor execution, than those during visual imagery [50]. All 4 MI trials were presented after the 4 ME trials to facilitate kinesthetic MI. Hence, the ME and MI trials were presented in a fixed order (first 4 trials ME, last 4 trials MI).

The data of the localizer session was analyzed offline using Brain Voyager QX v.2.3.1 and used to identify the region of interest (ROI) for the NF task, which was performed during the second session. The ROI for the NF task was extracted individually for each participant. T1 images were transformed to AC-PC space and Talairach space. Functional images were realigned using rigid-body affine transformations and coregistered automatically with the T1 scans. As pointed out by Söros et al., a cluster of activation in the left precentral cortex could be observed [4]. With the purpose of delivering feedback, activation clusters in this structure were determined in each participant using a statistical threshold of *t* > 3.8. Clear clusters of activity in the left lateral precentral gyrus were observed in all participants during the MI localizer task. The Talairach coordinates of the ROI were determined for the contrast MI_Offline > Rest (see results section for more details). The selected ROI was stored and used as feedback region for the subsequent NF training session.

### Session 2: fMRI During Neurofeedback

During the second session, participants received real-time feedback of activation changes in the left lateral precentral gyrus using Turbo-BrainVoyager (TBV; Brain Innovation, The Netherlands). The sessions started with a T1 scan. The T1 scan of the second session was coregistered with the T1 scan of the first session using BrainVoyager QX v.2.3.1 and the resulting transformation matrix was stored for later use in Turbo Brain Voyager.

The NF task consisted of two separate runs. Between the runs, participants stayed in the scanner for a short break (about 2 min). Each run included 10 resting trials and 10 NF trials. All trials had the same duration of 30 s. Visual feedback about the level of activation changes in the feedback ROI was provided by a moving gray thermometer bar presented on a screen, which was seen by the participant via a mirror attached to the head coil inside the scanner. The feedback value was calculated with the following formula:

*fb* = (*val* − *bl*)/*bl* × 100 (Turbo-BrainVoyager, Brain Innovation, The Netherlands).

**Fig. 2:**
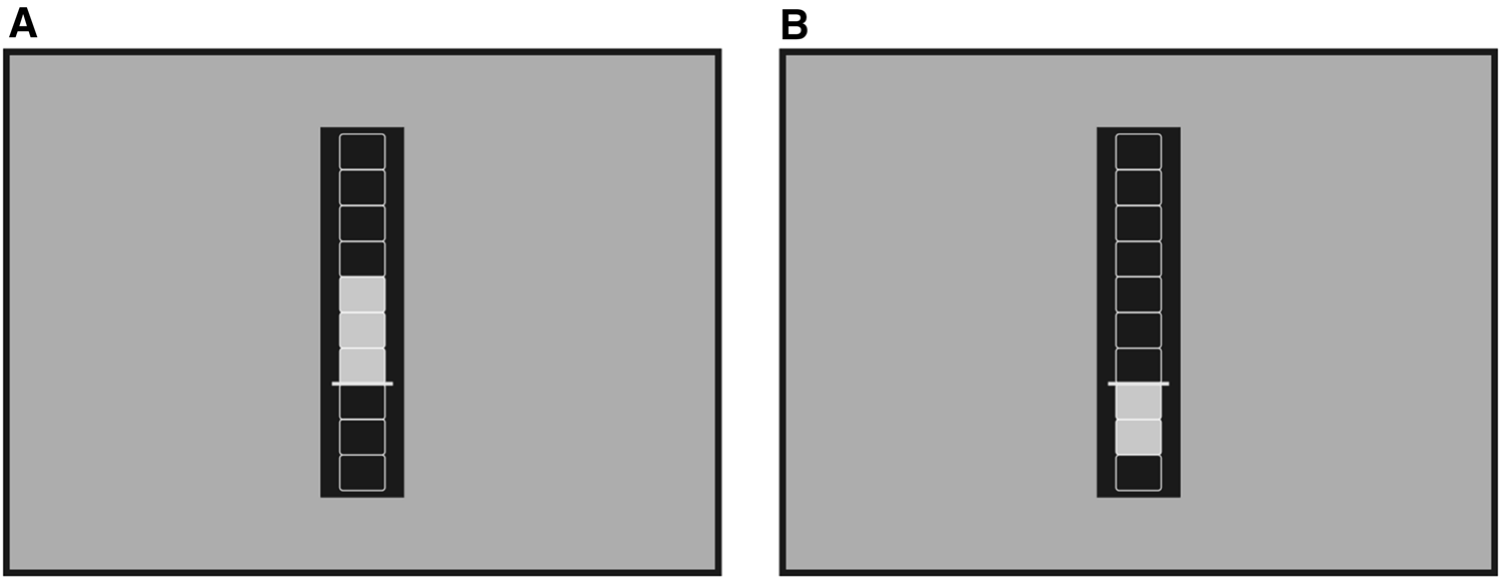
Feedback screen. The yellow horizontal line indicates the baseline threshold, which was set to 30%. **a** Activation above the baseline filled the upper part of the thermometer with gray color (four of the seven boxes above the threshold were filled with gray color if the feedback value was 1.14%). **b** Activation below the baseline filled the lower part of the thermometer with gray color (two of the three boxes below the threshold were filled with gray color if the feedback value was − 0.57%)

The feedback value (fb) represented a percent signal value and was used to fill the feedback thermometer. The value (val) stood for the mean activation of all voxel in the feedback ROI (in our case the left lateral precentral gyrus) at the current time point. To calculate the baseline (bl), we used the resting trial directly before each feedback trial to avoid global signal drifts. To consider the hemodynamic delay, we used a shift of three time points at the beginning and one time point at the end of each baseline condition. For the feedback presentation, we set the baseline threshold to 30% of the thermometer. Therefore, a yellow horizontal line was set at 30% of the thermometer height to indicate the threshold (Fig. 2). Activation above the baseline filled the upper part of the thermometers (parts above the threshold, Fig. 2a) whereas activation below the baseline filled the lower part (parts below the threshold, Fig. 2b). How much of the thermometer was filled by the fb was determined by the maximum percent signal value (Max PSC 1), which we set to 2%. The thermometer bar above the threshold was split up in seven boxes (Fig. 2). Three boxes were depicted below the threshold (Fig. 2). If the fb was 2%, all seven boxes above the threshold were filled with gray color. If the fb was 1%, three of the seven boxes above the threshold were filled with gray color (Fig. 2a). If the fb was 1.14%, four of the seven boxes above the threshold were filled with gray color. If the fb was − 1%, all three boxes below the threshold were filled with gray color. If the fb was − 0.57%, two of the three boxes below the threshold were filled with gray color (Fig. 2b). Furthermore, we used the average feedback values option to average the previous two time points with the current time point to calculate the current fb. This was used to stabilize the feedback signal within each NF trial.

During the NF trials, participants were instructed to increase the activation in the ROI by means of MI of swallowing movements. The instruction on how to imagine swallowing movements was the same as in the first session. The task was to increase the height of the thermometer bar above the threshold. Participants were instructed that the height of the thermometer bar reflected their brain activity in brain areas, which are active during executing swallowing movements. They were also instructed that they can voluntarily modulate this brain activity and consequently the height of the thermometer bar by imagining swallowing movements. During the resting trials, participants were instructed to stop imagining swallowing movements and to relax so that the thermometer bar decreases again. Participants were instructed to avoid active swallowing movements during the whole NF task.

### MRI Data Acquisition

We used a 3.0 T Siemens Skyra MRI scanner at the MRI-Lab Graz (Austria http://bioimaginggraz.at/). Participants were positioned comfortably in a supine orientation with their head located in a 32 channel head coil for signal reception. Functional images were acquired using a T2* weighted gradient-echo pulse imaging sequence (TR = 2400 ms; TE = 30 ms; flip angle = 90◦; matrix = 68 × 68; slice thickness = 3.5 mm; voxel dimensions = 3.5 × 3.5 × 3.5 mm) providing whole brain coverage in 36 slices. Anatomical images were recorded using a T1-weighted MPRAGE sequence (TR = 2530 ms; TE = 2.26 ms; flip-angle = 9°; slice thickness = 1 mm; 256 × 256 acquisition matrix; voxel dimensions = 1 × 1 × 1 mm; TI = 900 ms). To minimize head movements during measurements, foam padding was used around the head within the head coil. In addition, participants were given ear plugs to reduce discomfort due to scanner noise. During fMRI scanning, participants viewed the experimental tasks on a screen, via a mirror attached to the head coil.

### fMRI Data Analysis

SPM 8 (http://www.fil.ion.ucl.ac.uk/spm/) was used to preprocess and analyze functional whole brain data with the purpose of analyzing group effects. Functional MRI data were realigned using the first scan as a reference to which all subsequent scans were realigned. A mean image was created from the realigned volumes and spatially normalized to the MNI EPI template brain as implemented in SPM8. The derived spatial transformation was then applied to the realigned T2* volumes, which were spatially smoothed to improve the quality of group level statistics with a Gaussian kernel of 8-mm FWHM [51].

The General Linear Model of SPM8 was used in a block design. Two design matrixes were used:

(i) to compare ME and MI during the first session, where no real-time feedback was provided, we used the following design matrix: motor execution (ME) versus motor imagery offline (MI_Offline).
(ii) to compare MI between the two NF runs performed during the second session (NF run 1 = NFR1 vs. NF run 2 = NFR2) as well as to compare MI during the first session, where no real-time feedback was provided (= MI_Offline), with the MI tasks during the two NF runs of the second session the following design matrix was used: MI_Offline versus NFR1 versus NFR2.

Following these preprocessing steps, whole-brain analysis of the following contrasts of interest was performed:

(i) for session 1, we compared the ME task with the resting condition (ME > Rest), we compared the MI task of session 1, where no real-time feedback was provided, with the resting condition (MI_Offline > Rest), and we directly compared the ME and the MI task of session 1 (ME > MI_Offline; ME < MI_Offline).
(ii) for session 2, we compared the first MI NF run with the resting condition (NFR1 > Rest), we compared the second MI NF run with the resting condition (NFR2 > Rest), we directly compared the two MI NF runs with each other (NFR1 < NFR2; NFR1 > NFR2), and we also compared the MI task of the first session, where no feedback was provided, with the average of both MI NF runs of session 2 (MI_Offline < NFR1&NFR2; MI_Offline > NFR1&NFR2).

Hence, activation patterns observed during ME and MI of swallowing and differences between MI and ME were investigated as well as activation patterns during NF trials and differences between the offline MI task and NF trials. All results were considered with a threshold of *p* < 0.05 FDR [false discovery rate (FDR)] corrected for multiple comparisons on cluster-level with a minimum cluster size of 20 voxels. In the second-level analysis, age and sex of participants were used as covariates [51]. All coordinates are reported in MNI space.

In addition, a ROI analysis was performed using the SPM toolbox MarsBaR [52]. Therefore, the beta weights of the cluster around the left lateral precentral gyrus, which was used as feedback ROI, were extracted for the contrasts MI_Offline > Rest, NFR1 > Rest, and NFR2 > Rest. The beta weights of these contrasts were then statistically compared using paired *t*-tests. Pearson’s correlations were calculated to reveal a possible relationship in activation patterns between these conditions.

## Results

### Session 1: Functional Localizer—Motor Execution Versus Imagery Offline

**Fig. 3:**
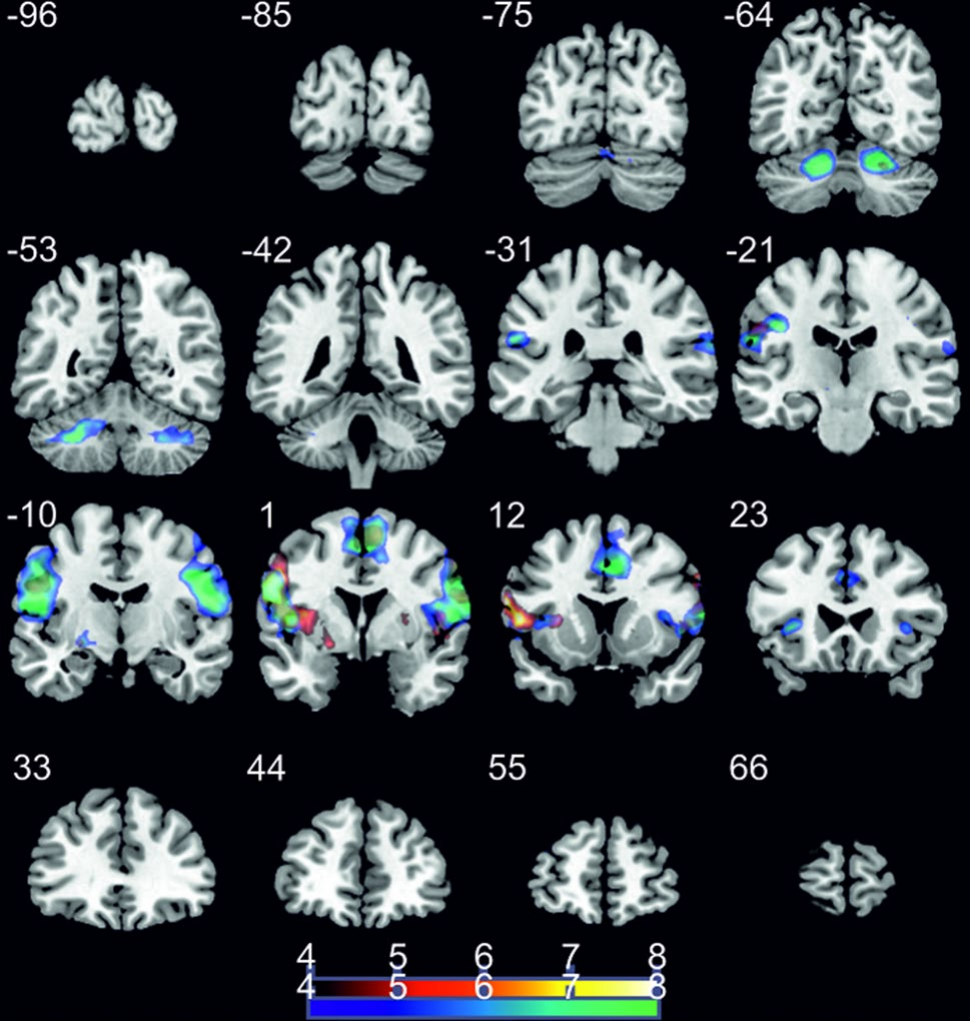
Coronal slices; *t*-score map for motor execution (cold colors) and motor imagery offline (hot colors) compared to rest; *p* < 0.05 corrected for multiple comparisons on cluster-level [false discovery rate (FDR)]; minimum cluster size 20 voxels; numbers next to the slices indicate the y coordinates of each slice

To examine brain activation patterns during ME and MI of swallowing, we contrasted both conditions with the resting condition. During ME of swallowing, a large network of brain areas was active including the bilateral cerebellum, bilateral pre- and postcentral gyrus, basal ganglia, the insula, motor areas and the SMA (Table 1). During MI of swallowing when no real-time feedback was provided (MI_offline), comparable brain areas were active than during ME of swallowing (Table 1). Figure 3 illustrates the activation patterns during MI and ME of swallowing during the localizer task compared to the rest condition.

**Table 1:** Brain regions (clusters) preferentially activated during ME of swallowing compared to rest and during MI of swallowing offline compared to rest

	Voxels	Peak			*T*-value	*Z*-score
		*x*	*y*	*z*		
*ME *> *rest*						
R cerebellum	695	20	− 64	− 24	13.28	4.89
L cerebellum	747	− 30	− 52	− 38	11.91	4.73
L globus pallidus	77	− 16	− 8	− 8	11.49	4.67
L lateral precentral gyrus	2895	− 54	− 4	30	21.97	5.62
L insula						
L inferior frontal gyrus						
L middle frontal gyrus						
L premotor cortex						
L primary motor cortex						
L postcentral gyrus						
R lateral precentral gyrus	2629	56	4	12	15.13	5.09
R insula						
R inferior frontal gyrus						
R middle frontal gyrus						
R premotor cortex						
R primary motor cortex						
R postcentral gyrus						
R insula	82	36	14	8	7.80	4.05
L & R SMA	1369	8	− 2	56	23.16	5.69
*MI *> *rest*						
R cerebellum	21	22	− 60	− 28	5.48	3.44
L Putamen	38	− 20	0	− 8	7.33	3.94
L globus pallidus						
R Putamen	40	26	6	4	7.42	3.96
L lateral precentral gyrus	1468	− 62	8	18	17.20	5.28
L insula						
L inferior frontal gyrus						
L middle frontal gyrus						
L premotor cortex						
L postcentral gyrus						
R lateral precentral gyrus	546	64	4	20	9.14	4.31
R insula						
R inferior frontal gyrus						
R middle frontal gyrus						
R premotor cortex						
R postcentral gyrus						
L & R SMA	205	10	− 2	60	6.98	3.86

Reported coordinates in MNI space; *L* left, *R* right; *p* < 0.05 corrected for multiple comparisons on cluster-level [false discovery rate (FDR)]; minimum cluster size 20 voxels

To reveal differences in activation patterns between ME and MI, we also contrasted ME and MI. Executing swallowing movements led to stronger bilateral activation patterns in the cerebellum, brainstem, calcarine cortex, pre- and postcentral gyrus, insula, and cingulate cortex (Table 2). In contrast, MI of swallowing led to stronger left-lateralized activation in the inferior frontal gyrus, amygdala, and inferior and superior parietal regions (Table 2).

**Table 2:** Brain regions (clusters) preferentially activated during ME of swallowing compared to MI of swallowing offline

	Voxels	Peak			*T*-value	*Z*-score
		*x*	*y*	*z*		
*ME *> *MI*						
L cerebellum	1817	− 26	− 58	− 40	15.92	5.16
L brainstem	30	− 12	− 20	− 4	6.74	3.80
L Calcarine	318	− 20	− 66	10	9.15	4.31
L lingual gyrus						
L postcentral gyrus						
L cuneus						
L posterior cingulate						
R Calcarine	709	20	− 48	8	8.07	4.10
R lingual gyrus						
R precuneus						
R cuneus						
R posterior cingulate						
R cuneus	67	20	− 80	46	9.98	4.45
L postcentral gyrus	1573	− 40	− 20	28	17.73	5.32
L insula						
L inferior frontal gyrus						
L premotor cortex						
L primary motor cortex						
L precentral gyrus						
L parietal lobe						
L temporal lobe						
R postcentral gyrus	2214	62	− 8	20	19.98	5.49
R insula						
R inferior frontal gyrus						
R premotor cortex						
R primary motor cortex						
R precentral gyrus						
R parietal lobe						
R temporal lobe						
L insula	190	− 36	− 26	18	13.69	4.94
R insula	25	36	16	8	7.73	4.03
R middle frontal gyrus	59	30	50	20	6.70	3.79
R cingulate gyrus	239	14	6	46	7.70	4.02
R SMA						
*ME *< *MI*						
L amygdala	24	− 30	− 2	− 28	7.71	4.03
L inferior frontal gyrus	20	− 44	38	16	5.91	3.57
L inferior parietal lobe	22	− 44	− 34	46	7.13	3.89
L superior parietal lobule	24	− 34	− 56	58	5.78	3.53

Reported coordinates in MNI space; L, left; R, right; *p* < 0.05 corrected for multiple comparisons on cluster-level [false discovery rate (FDR)]; minimum cluster size 20 voxels

### Session 2: Neurofeedback Task

First, we compared the NF runs with the resting condition. During both NF runs, brain areas, which were associated with active swallowing in prior fMRI studies, were active (Table 3).

**Table 3:** Brain regions (clusters) preferentially activated during the first and the second run during the NF task compared to rest

	Voxels	Peak			*T*-value	*Z*-score
		*x*	*y*	*z*		
*NF Run1* > *rest*						
R cerebellum	131	28	− 46	− 42	5.88	3.56
L cerebellum	117	− 20	− 62	− 24	7.34	3.94
R globus pallidus	57	16	0	− 6	7.44	3.97
L globus pallidus	55	− 16	0	− 10	13.79	4.95
L lateral precentral gyrus	1329	− 50	− 4	16	13.32	4.90
L insula						
L inferior frontal gyrus						
L middle frontal gyrus						
L premotor cortex						
L primary motor cortex						
L postcentral gyrus						
R lateral precentral gyrus	794	62	4	36	16.50	5.21
R inferior frontal gyrus						
R middle frontal gyrus						
R premotor cortex						
R postcentral gyrus						
L & R SMA	424	8	− 2	60	7.57	3.99
*NF Run2* > *rest*						
R cerebellum	93	30	− 74	− 28	7.60	4.00
L cerebellum	133	− 16	− 62	− 24	6.18	3.65
L lateral precentral gyrus	265	− 62	4	28	8.14	4.12
L inferior frontal gyrus						
L premotor cortex						
L postcentral gyrus						
R lateral precentral gyrus	175	60	2	36	7.17	3.90
R inferior frontal gyrus						
R premotor cortex						
R postcentral gyrus						
L postcentral gyrus	159	− 46	− 6	18	10.30	4.50
L insula						
L SMA	22	− 6	2	64	4.84	3.22

Reported coordinates in MNI space; *L* left, *R* right; *p* < 0.05 corrected for multiple comparisons on cluster-level [false discovery rate (FDR)]; minimum cluster size 20 voxels

When comparing the first (NFR1) and the second NF run (NFR2), only the right precuneus showed a stronger activation during the second compared to the first run (voxels: 21, peak: *x*: 2, y: − 64, *z*: 54, *T*-value: 5.94). No significant activation differences could be observed in the contrast NFR1 > NFR2. Since there were no prominent differences in brain activation patterns between the two NF runs, we compared the MI offline task of the first session with both NF runs (MI_Offline < NFR1&NFR2; MI_Offline > NFR1&NFR2).

The contrast MI_Offline > NFR1&NFR2 revealed no significant results.

**Fig. 4:**
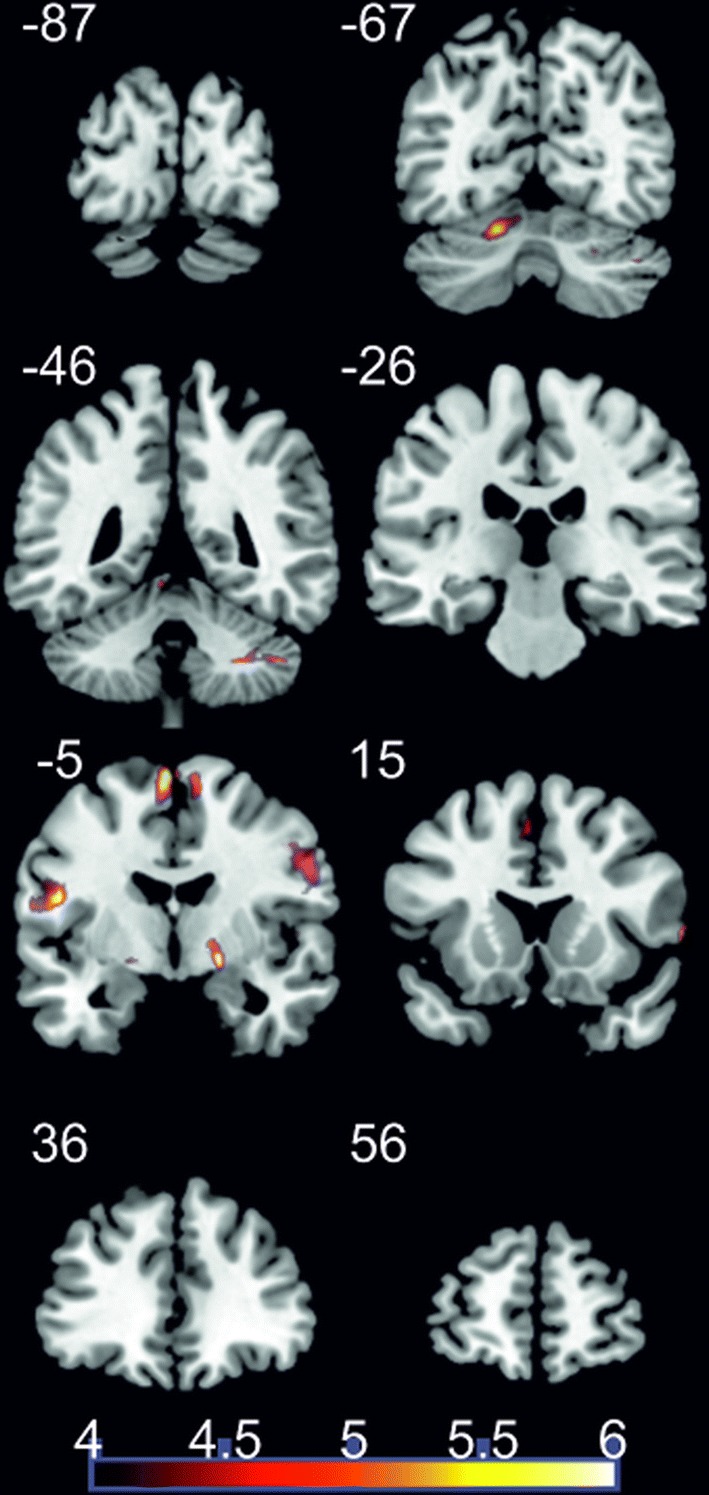
Coronal slices; *t*-score map showing stronger activation during the NF runs compared to the MI offline task; *p* < 0.05 corrected for multiple comparisons on cluster-level [false discovery rate (FDR)]; minimum cluster size 20 voxels; numbers next to the slices indicate the y coordinates of each slice

During the NF runs, bilateral brain regions, which are involved in the swallowing process, including the cerebellum, pre- and post-central regions, SMA, basal ganglia, as well as visual brain regions were stronger activated compared to the MI offline task (Table 4, Fig. 4).

**Table 4:** Brain regions (clusters) preferentially activated during the first and second NF run compared to the MI offline task

	Voxels	Peak			*T*-value	*Z*-score
		*x*	*y*	*z*		
*MI offline* < *NF Run 1 & 2*						
R cerebellum	86	24	− 44	− 44	7.00	3.86
L cerebellum	78	− 16	− 62	− 18	7.16	3.90
R globus pallidus	48	18	− 4	− 8	7.25	3.92
L occipital lobe	29	− 30	− 100	12	6.35	3.69
L superior occipital lobe	51	− 12	− 100	22	7.65	4.01
L lateral precentral gyrus	66	− 54	8	42	6.21	3.66
L inferior frontal gyrus						
L premotor cortex						
R lateral precentral gyrus	250	62	2	38	6.85	3.83
R inferior frontal gyrus						
R middle frontal gyrus						
R premotor cortex						
R postcentral gyrus						
L postcentral gyrus	56	− 48	− 6	18	6.63	3.77
L SMA	192	− 4	− 6	68	6.67	3.78
R SMA	51	8	2	64	5.39	3.41

Reported coordinates in MNI space; *L* left, *R* right; *p* < 0.05 corrected for multiple comparisons on cluster-level [false discovery rate (FDR)]; minimum cluster size 20 voxels

**Fig. 5:**
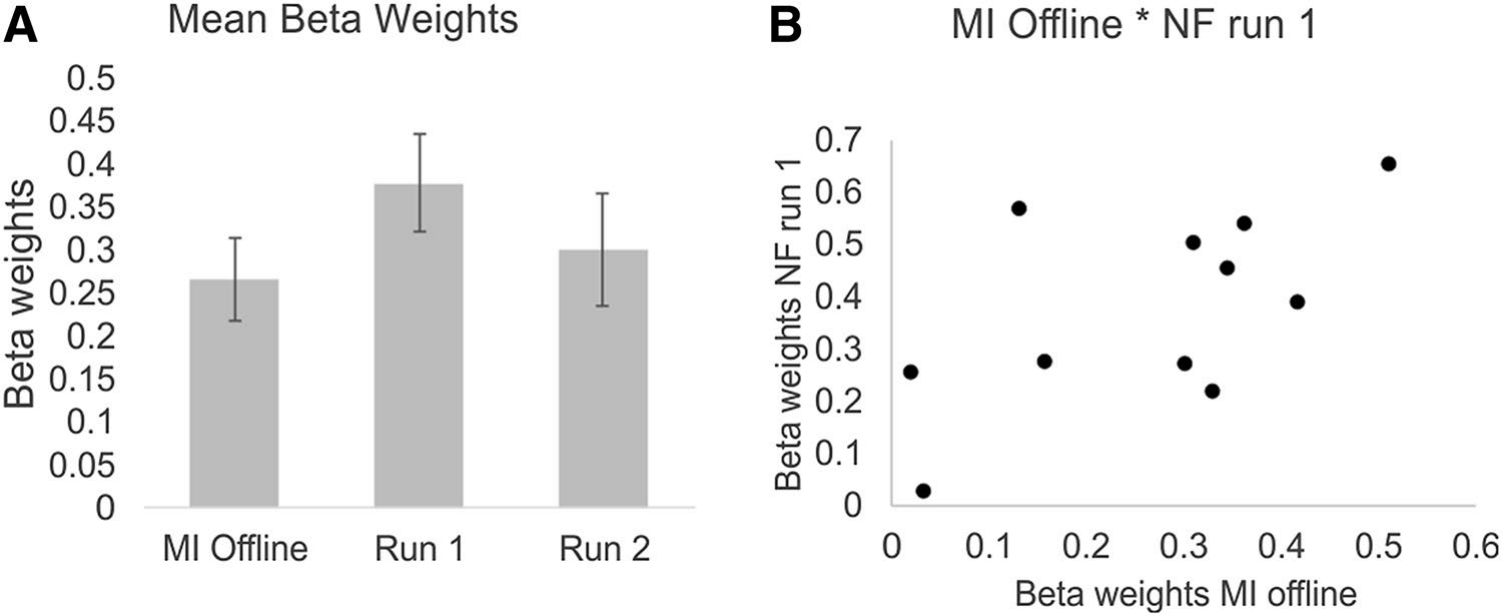
**a** Beta weights (means and *SE*, beta weights were extracted for the contrasts MI_Offline > Rest, NFR1 > Rest, and NFR2 > Rest) of the feedback ROI (left precentral gyrus) for the MI offline task and the two NF runs. **b** Correlation between beta weights extracted from the feedback ROI during the MI offline task and the first NF run

When comparing the beta weights in the feedback ROI (left lateral precentral gyrus) between conditions (beta weights for the contrasts MI_Offline > Rest vs. NFR1 > Rest vs. NFR2 > Rest), activity in the feedback ROI was significant higher during the first NF run compared to the MI offline task (*t* (10) = − 2.42, *p* < 0.05, Fig. 5a). In the second feedback-trial run, activity in the ROI was numerically lower than during the first run; however, this difference did not reach statistical significance (*t*(10) = 1.12, *ns.*, Figure 5a).

Activity levels observed during the MI offline task and the first feedback run in the feedback ROI correlated significant positively (*r* = 0.61, *p* < 0.05, Fig. 5b). Hence, the higher the activation during the offline MI task the higher the ability to upregulate activation during the first feedback run. A significant correlation in the activation level in the feedback ROI could be found neither between run 1 and run 2 nor between run 2 and the MI offline task.

## Discussion

In the present study, we first investigated whether MI and ME of swallowing lead to comparable brain activation patterns. Second, we performed a real-time fMRI NF training in which healthy individuals were instructed to increase the activity in brain regions, which are involved in active swallowing, while imagining swallowing movements.

### Motor Execution Versus Imagery of Swallowing—Offline Task

#### Motor Execution Versus Rest

In line with prior fMRI studies, ME of swallowing led to increased activity in many different brain regions. During motor execution, we found strong bilateral activation in the lateral precentral gyrus. This cluster included the insula, inferior frontal gyrus, middle frontal gyrus, the premotor and primary motor cortex, and parts of the postcentral gyrus.

Our findings are in line with prior studies that investigated neuronal correlates of ME of swallowing. The most consistent and prominent region of activation reported in prior studies corresponds to the lateral precentral gyrus including the primary motor cortex and the premotor cortex [3, 4, 53]. The oral, pharyngeal, and esophageal musculature [53] involved in voluntary active swallowing [5, 6, 54, 55] are represented in the precentral cortex [3].

Lateral parts of the postcentral gyrus, which correspond to primary somatosensory cortex of the face, are also involved in executing swallowing movements [3]. This area is generally involved in a variety of related functions including the processing of sensory stimuli applied to the face [56] as well as taste sensation [3].

In the present study, insula activation was found bilaterally within the precentral gyrus cluster but also an extra cluster was found for the right insula during ME. The insula receives afferent inputs from and also projects to many different brain regions, which are active during swallowing, and is strongly involved in the swallowing process [3, 4, 6, 57, 58]. In addition, the insula is assumed to represent a primary gustatory cortex [3, 59]. Hence, the insula seems to be involved in sensory and motor processes regulating active swallowing. In humans, damage to the insula and inner side of the operculum often leads to dysphagia underlining the importance of these brain regions for the swallowing process [60, 61].

Swallowing also activated the bilateral SMA, which is in line with prior findings [4]. It is assumed that active swallowing leads to similar brain activation patterns than speech production, including SMA and the gyrus cinguli [4]. These areas are implicated in the programming, execution, and control of fine, sequential movements [62, 63]. The SMA is activated during whistling, chewing, and tongue movements [4, 64]. It seems to play an important role in the preparation of volitional swallowing, possibly in conjunction with inputs from dorsal prefrontal cortex and insula [6].

In a meta-analysis, Sörös et al. report on activation patterns in the inferior frontal gyrus and the middle frontal gyrus during saliva swallowing comparable to the present findings. They also found activation in the basal ganglia, but in contrast to the present findings, they found an involvement of the putamen but not of the globus pallidus during active saliva swallowing [4]. However, in line with the present findings, they also report the involvement of the basal ganglia in the left hemisphere, but not in the right hemisphere during execution of swallowing movements. It is assumed that active saliva swallowing is mediated by a basal ganglia-thalamocortical motor circuit [4, 65, 66].

Previous neuroimaging studies recording data with a field of view covering the cerebellum reported consistently the involvement of the cerebellum in active swallowing [4–6, 67, 68] as well as in other orofacial movements—lip and tongue movements, and whistling [69–72]. In general, during movement preparation the brain does not only send motor commands to muscles. A copy of these signals is also send to the cerebellum. It is assumed that sensory consequences of the forthcoming motor action are predicted by cerebellar neural circuits. The expected sensory consequences are then compared to actual sensory feedback from the periphery. Based on this comparison, error signals are computed and relayed to different cortical areas. This update ultimately allows for a fast and adaptive movement performance without delays [73]. This cortico-cerebellar loop is an established concept in motor control theory and also account for executing swallowing movements.

#### Motor Imagery Versus Rest

Comparably to studies investigating MI and ME of hand and foot movements, MI of swallowing activated a similar network of brain areas than executing swallowing movements [40, 41]. This is also in line with findings of prior NIRS studies that investigated MI and ME of swallowing movements [44–46]. In these NIRS studies, MI of swallowing led to the strongest hemodynamic response over the bilateral inferior frontal gyrus, around Brodmann area (BA) 44 [44–46]. In the present study, when contrasting the MI offline task with the resting condition the two largest clusters of activation were located around the left and right lateral precentral gyrus. These clusters also included the inferior frontal gyrus. In addition, NIRS studies report on activation in premotor areas and SMA during MI of swallowing, which is also in line with the present findings [44–46]. However, because of the limited spatial resolution and the restriction to the assessment of brain activity in the outer layer of the cortex [48, 49], prior NIRS studies cannot reveal all neuronal correlates of MI of swallowing. Here, we showed that MI of swallowing also activated brain structures, which are located deeper in the brain such as the insula, basal ganglia, and cerebellum.

#### Motor Execution Versus Motor Imagery

Overall, executing swallowing movements led to a stronger activation in the neuronal network, which is associated with active swallowing (including the pre- and postcentral gyrus, inferior and middle frontal gyrus, premotor and primary motor areas, SMA, insula, cerebellum, basal ganglia), than MI of swallowing. This is in line with prior MI/ME studies, which also found a stronger activation during ME of different movement tasks in classic sensorimotor regions compared to MI [35, 36, 38, 40, 41, 44–46].

During ME of swallowing, the brain stem and the closely connected cerebellum were more active compared to MI. These areas are more involved in reflexive phases of the swallowing process and less involved in voluntary phases, which might explain the stronger activation during ME than during MI [1].

The cingulate gyrus and temporal areas were also more strongly involved in ME than in MI of swallowing. These brain areas are often associated with ME of swallowing. Prior studies report on activation in the anterior cingulate cortex (ACC) during active swallowing but also during other orofacial motor behaviors performed, such as tongue protrusion, suggesting the possibility of a more basic role in orofacial function [3, 6, 74].

The cluster termed “Calcarine” (Table 2) includes different brain regions such as the postcentral gyrus, cuneus, precuneus, or posterior cingulate. These areas are likely to have a sensory role in the control of swallowing [6]. For instance, the precuneus and posterior cingulate cortex are considered as association areas with rich reciprocal connections to the thalamus. Consequently, they are thought to play a role in integrating sensory information during active swallowing [6]. These areas might be involved in the reception and higher processing of sensation arising from the oropharynx and esophagus [6]. They are reciprocally connected with motor areas in the frontal lobe [75]. Activation of theses more posterior areas may represent the integration of thermal, gustatory, and somatosensory information during active swallowing [4, 6]. Such somatosensory information is lacking for the MI task.

During MI, we found a stronger activation in the inferior frontal gyrus, the inferior and superior parietal lobule, and the amygdala in comparison to the ME task. In general, the imagination of a movement involves metacognitive processes such as focusing attention on inner states as well as introceptive and self-referential processes. These processes are also involved in NF control [76]. During NF training, a network of brain areas is active, which is associated with self-referential processes such as focusing attention to inner states [77]. This network includes the inferior frontal gyrus as well as the inferior and superior parietal lobule [78, 79]. This network might be also involved in the offline MI task. A meta-analysis by Hardwick et al. (2017) also showed that a large area of the parietal cortex, spanning the inferior and superior parietal lobule, was more consistently associated with MI than ME of hand, foot, and face movement tasks [40]. Hence, a stronger activation in the inferior frontal gyrus and the inferior and superior parietal lobule during MI compared to ME of swallowing might be related to higher cognitive processes, which are stronger involved in MI.

The amygdala is strongly connected with the insula and may therefore play a role in the swallowing process [5]. Furthermore, the amygdala is involved in emotional self-regulation [80]. Marins et al. found an involvement of the amygdala during MI of hand movements, too. However, this was also evident in the ME task [81].

### Real-Time fMRI Neurofeedback

#### Neurofeedback Runs Versus Rest

As feedback region, we chose the left lateral precentral gyrus because this region was active in all participants during the functional localizer task. During the NF runs, similar brain regions were activated than during ME of swallowing as well as during the MI offline task. Hence, while receiving real-time feedback, the same brain network was active than during the offline tasks.

#### Motor Imagery Offline Versus Neurofeedback Runs

During the NF runs, brain activity in the feedback ROI was significantly higher than in the MI offline task. Hence, when receiving real-time feedback of the activation level in brain regions, which are involved in the swallowing process, participants were able to increase this brain activation voluntarily while imagining swallowing movements. This is in line with a prior NIRS-based NF study, in which healthy participants were also able to voluntarily modulate the activity in the inferior frontal gyrus during MI of swallowing [45]. Future studies should address the question whether repeated NF training sessions can also lead to functional and structural changes in the brain network associated with swallowing.

Activity in the feedback ROI during the first NF run was slightly higher than during the second one, although this difference did not reach significance (Fig. 5a). The numerically lower activation during the second run might have been caused by an increased fatigue over the training course. After the NF session, participants also subjectively reported that their level of concentration decreased from the first to the second run. A more interesting and motivating design of the feedback screen might be useful in future studies to reduce possible decreases in motivation and/or concentration [48, 82]. Probably, a stronger NF training effect, e.g., indicated by a linear increase in the activation level of the feedback ROI over time, might be reached by providing alternative NF instructions. There is evidence that providing feedback without explicit instructions to use specific mental imagery strategies enables more effective learning [33, 83, 84].

During the NF training runs, stronger activation not only in the feedback ROI (left lateral precentral gyrus) but also in other brain regions associated with swallowing was observed in comparison to the MI offline task. NF studies that provided feedback from intra-cell recordings showed that it is possible to modulate activity in specific brain regions [33]. However, during real-time fMRI studies using MI of limb movements as mental strategies, an activation in a larger brain network exceeding the feedback ROI is often reported [81, 85, 86]. During NF control, higher cognitive control mechanisms are recruited [33, 76, 78, 79, 87, 88], which might also explain activation patterns outside the feedback ROI observed during NF training. Brain areas associated with higher cognitive control mechanism during NF include among others the inferior frontal gyrus, anterior insula, the cingulate cortex, basal ganglia, SMA, lateral prefrontal areas, the inferior and superior parietal lobule, as well as the occipital cortex [78, 79, 87]. The stronger activation in the occipital lobe during the NF runs compared to the MI offline task might be also explained by the visual feedback provided during NF, while in the MI offline task no moving objects but only static instructions (3 s.) followed by a fixation cross (27 s.) were presented.

We also observed a positive correlation between the activity in the feedback ROI assessed during the MI offline task and the first feedback run. The higher the activation during the offline MI task the higher the activation during the first feedback run. Hence, the activity observed in the localizer task might predict the subsequent NF performance. A substantial proportion of NF users (up to 30%) fail to self-regulate specific brain activity and the reasons for this inability to modulate one’s own brain activity are largely unknown. Therefore, an increasing number of studies try to find possible predictors of successful NF modulation [33, 76, 82, 84, 88–90]. For instance, there is evidence that brain structure can predict the NF performance [87, 88, 91, 92] but also brain activity observed during resting measurements can be a predictor [93, 94]. Activity in the feedback ROI observed during the MI offline task and the second NF run did not correlate significantly. This may be due to the fact that during the second run the overall activation level was slightly reduced compared to the first run. A reduction in the activation in the feedback ROI during the second run compared to the first run might be a result of a decrease in motivation and concentration over time as outlined above. Nevertheless, our results indicate that a higher BOLD response in the feedback ROI during the localizer task might increase the probability that the participant can voluntarily modulate the activity in this feedback ROI during NF training.

## Limitations and Future Directions

In the present investigation, we only compared neuronal correlates of MI and ME of swallowing saliva. Since there is evidence that swallowing saliva can elicit different brain activation patterns than swallowing water or barium [4, 54, 64, 95–98], it would be interesting to investigate whether executing and imagining swallowing of another bolus type also leads to comparable brain activation patterns such as executing and imagining swallowing of saliva. Furthermore, there is evidence that older adults recruited more cortical regions than young adults during active swallowing, such as the inferior frontal gyrus pars opercularis [95]. Hence, the present finding in young individuals has to be replicated in older individuals, since dysphagia is mainly prevalent in older people [8, 10–12].

A limitation of the present study is that we could not record how many swallows the participants actually performed during the ME task or how many swallows they imagined during the MI task. Furthermore, participants were instructed to avoid active swallowing during the MI task; however, we could not control for actual swallows during this imagery task. In general, monitoring active swallowing behavior while participants are lying in an fMRI scanner is possible [95]. However, it is not possible to measure the amount of imagined swallows, when participants should not move. Therefore, we instructed the participants to swallow in a regular, self-paced rhythm during the 30 s ME trials, which resulted in approximately 5–6 swallows per trial (this was observed during a practice trial before participants were laid in the scanner). For the MI task, participants were instructed to imagine swallowing in the same rhythm as was done during the ME task. This practice trial was performed before the start of the first fMRI session. Nevertheless, we cannot exclude that different frequencies of swallowing contribute to different activation patterns. However, brain activation patterns between individual participants were largely comparable, indicating that their swallowing behavior in the scanner was comparable, too.

Our results have practical implications for the use of NF training to treat symptoms of dysphagia in the future. Because of the high prevalence of dysphagia and the need for new dysphagia treatments [10–15, 61], NF might be an adequate method directly addressing the neuronal basis of swallowing [33, 34]. For instance, dysfunctional brain activation patterns after a brain lesion causing swallowing problems might be restored by using NF. MI of swallowing might lead to neuronal plasticity processes and thereby improve swallowing function [25–34]. In this context, there is evidence that external stimulation of the swallowing motor cortex using repetitive transcranial magnetic stimulation (rTMS) leads to recovered swallowing function in patients with dysphagia [99, 100]. Using NF applications, participants can learn to modulate voluntarily the activation level in specific brain areas of the swallowing network, without the need of external stimulation such as rTMS [46].

## Conclusion

We successfully showed that ME and MI of swallowing movements led to comparable brain activation patterns in the whole brain. In addition, we could show that healthy individuals are able to increase voluntarily the activity in brain regions, which are generally active during executing swallowing movements, during NF training by means of MI of swallowing. The present findings lay the foundation for future studies, in which (i) neuronal correlates of MI and ME of swallowing in dysphagia patients should be investigated [44] and (ii) effects of NF training using MI of swallowing on swallowing function in dysphagia patients need to be evaluated [44–46, 101].
